# Extracellular Vesicles as Drivers of Immunoinflammation in Atherothrombosis

**DOI:** 10.3390/cells11111845

**Published:** 2022-06-05

**Authors:** Rosa Suades, Maria Francesca Greco, Teresa Padró, Lina Badimon

**Affiliations:** 1 Cardiovascular ICCC Program, Research Institute Hospital de la Santa Creu i Sant Pau, IIB-Sant Pau, 08025 Barcelona, Spain; rsuades@santpau.cat (R.S.); mgreco@santpau.cat (M.F.G.); tpadro@santpau.cat (T.P.); 2 Department of Pharmacological and Biomolecular Sciences, Università degli Studi di Milano, 20133 Milan, Italy; 3 Centro de Investigación Biomédica en Red Cardiovascular (CIBERCV), Instituto de Salud Carlos III, 28029 Madrid, Spain

**Keywords:** atherothrombosis, atherosclerosis, extracellular vesicles, immunoinflammation, immunothrombosis, inflammation, microvesicles, thromboinflammation, thrombosis

## Abstract

Atherosclerotic cardiovascular disease is the leading cause of morbidity and mortality all over the world. Extracellular vesicles (EVs), small lipid-bilayer membrane vesicles released by most cellular types, exert pivotal and multifaceted roles in physiology and disease. Emerging evidence emphasizes the importance of EVs in intercellular communication processes with key effects on cell survival, endothelial homeostasis, inflammation, neoangiogenesis, and thrombosis. This review focuses on EVs as effective signaling molecules able to both derail vascular homeostasis and induce vascular dysfunction, inflammation, plaque progression, and thrombus formation as well as drive anti-inflammation, vascular repair, and atheroprotection. We provide a comprehensive and updated summary of the role of EVs in the development or regression of atherosclerotic lesions, highlighting the link between thrombosis and inflammation. Importantly, we also critically describe their potential clinical use as disease biomarkers or therapeutic agents in atherothrombosis.

## 1. Introduction

From the initial seminal discoveries [1,2] to the current state of research, the understanding of extracellular vesicles (EVs) has massively progressed and further advancements in the area are expected [3]. EVs are small lipid-bilayer vesicles that contain a complex cargo of nucleic acids, proteins, and lipids [4]. They are considered cell-to-cell signaling modules released from any cell type (both prokaryotic and eukaryotic) into distinct interstitial compartments and body fluids with potential value as disease biomarkers and drug-delivery vehicles [5]. Interestingly, EVs exert many biological processes contributing to health and disease homeostasis [6,7] and cardiovascular disease has become a fast expanding field of interest within EVs.

Atherosclerotic cardiovascular disease (ASCVD) remains the leading cause of death and disability worldwide and atherothrombosis underlies the majority of cardiovascular events [8]. Atherosclerosis chronically develops from early fatty streak to atheromatous plaque formation that when ruptured leads to platelet activation and, ultimately, thrombus formation [9]. We now acknowledge that atherosclerosis is indeed an inflammatory condition [10]. Understanding the clinical benefits of lipid-lowering therapies, such as statins, and other novel pharmacological approaches has provided evidence on the role of inflammation in all the stages of atherosclerosis. Similarly, EVs have also been related to all phases of the atherosclerotic process from initiation to unforeseen thrombotic complications [11]. In the present review, we explore the latest findings on EV-mediated mechanisms and their potential as diagnostic and prognostic biomarkers as well as novel therapeutic targets in inflammatory atherothrombosis.

## 2. Extracellular Vesicles: Current State and Emerging Concepts

Extracellular vesicles have been recognized as potential bioactive effectors with important roles in cell–cell communication involved in the regulation of a range of biological processes [6,7]. Distinct stimuli can trigger its release by cell activation and apoptosis or senescence under inflammatory, hypoxic, ischemic, or oxidative conditions, and also by mechanical stress such as high blood shear rates [4]. Once secreted, extracellular and pericellular matrix influence EV spreading locally and remotely via the bloodstream [12]. EVs are found ubiquitously in all human extracellular body fluids, such as plasma and urine, and tissues as well as in cell culture conditioned media. Their levels are the result of a fine balance between their shedding and clearance, in which different mechanisms intervene. EVs conform to a highly complex population demonstrated by high heterogeneity in size, biogenesis, origin, content, mechanism of action, and biological function. Indeed, their biogenesis, uptake by target cells and cargo delivery processes (endocytosis, trafficking to endo/lysosomes, membrane fusion, cargo release, and phenotypic changes in the recipient cell) are still unclear (please refer to the comprehensive review for detailed EV basic biology [13]). Mass spectrometry-based proteomics have shown that cargo selection mechanisms are highly regulated by post-translational modifications [14].

The fact that EVs are formed by several but not yet completely known mechanisms convolutes their classification. Following the foundation of the International Society for Extracellular Vesicles (ISEV) one decade ago, the generic ‘extracellular vesicle’ term was adopted to encompass all the distinct names used depending on their source, scale, density, formation, and content [15]. Thus, EVs can be characterized into different subtypes either exosomes, microvesicles (MVs), and apoptotic bodies or small, medium, and large extracellular vesicles, ranging from 20 nm to 5 µm. Briefly, apoptotic bodies (ApoBDs), also known as apoptotic extracellular vesicles (ApoEVs), are large vesicles (>1 µm) with apoptotic nuclear material and permeable membrane formed after cell shrinkage as a result of apoptotic cell death. Despite limited studies on their biological roles, ApoBDs presumably control the transfer of clearance signals among damaged cells. Microvesicles are small particles (100 nm–1 µm) that outwardly bud off from the cellular membrane, predominantly upon cytoskeleton remodeling and externalization of phosphatidylserine (PS), without a nucleus and harboring parental cell-specific surface antigens. They have been also referred to as microparticles, exovesicles, ectosomes, migrasomes, oncosomes, and prostasomes, among others, on the basis of their size and site or context of release. In contrast, nano-sized exosomes (30–100 nm; EXOs), also named exovesicles or endosomes, are endosomal intraluminal vesicles secreted when intracellular multivesicular bodies fuse with the plasma membrane. Multiple pathways have been identified in the regulation of exosome formation [16]. Finally, newly discovered EV subtypes named ‘exomeres’ and ‘supermeres’ have recently revolutionized the field [17,18]. These are described as non-membranous vesicles with a size of ≤50 nm bearing lipid and protein contents. However, there is still debate on whether they should be considered as EVs or extracellular particles like lipoproteins. Given the increasing relevance of EVs, it becomes crucial to uncover the precise function of each vesicle subtype. The following review will specifically report on MVs or large EVs due to their relevant role in ASCVD. Despite the review mainly focusing on MVs, we designate them as EVs throughout the manuscript and only use ‘MV’ or ‘large EVs’ terms to refer to those studies clearly reporting MV-associated data (with evidence of specific markers or subcellular origin). It is important to bear in mind that articles often refer to a mixed population of EVs due to their overlapping size and composition. ApoAB and EXO effects on atherosclerosis are reviewed elsewhere [19,20,21] and will not be the main focus of this review.

One of the most fascinating roles of extracellular vesicles is to engage in cellular crosstalk via the transfer of selective biomolecular cargo to recipient cells in an autocrine, paracrine, or endocrine manner to regulate cell function [22]. The effects of EVs on target cells are highly influenced by the parental cell, stimuli, local environment and, especially, composition. EVs are natural carriers of a highly diverse spectrum of bioactive molecular effectors mostly comprising lipids, proteins, and nucleic acids. In fact, there are several web-based resources and databases available to dig into these vesicular molecules such as Vesiclepedia, ExoCarta, EVpedia, exoRBase, EV-TRACK platform, and exRNA atlas. By means of lipidomics studies, EVs have shown to be highly enriched in cholesterol, eicosanoids, fatty acids, sphingomyelin, glycosphingolipid, phosphatidylserine, and other bioactive lipids, captured from the cytosol during EV formation or synthesized by EV-metabolizing enzymes. Membrane lipids are organized in a bilayer structure with lipid carriers such as fatty acid binding proteins and have the ability to confer a procoagulant surface. Proteomic and enrichment studies have enabled the unveiling of both membrane-bound and intraluminal soluble proteins in EVs. Although a truly selective and universal marker of EV types is not well-defined yet, specific proteins have been used to distinguish EV subtypes such as Annexin A1, identified as specific marker for MVs shed directly from the plasma membrane [23]. Similarly, cell surface-specific markers (cluster of differentiation (CD) markers) are widely used to characterize different EV subpopulations [24]. It has been debated whether these markers are always reflective of their true cell origin or have just attached to the EV membrane. Indeed, recent findings described that EVs themselves transport extracellular proteins interacting with their plasma membrane named protein corona and that EV-associated proteins could also have a pathophysiological relevance and, therefore, should not be considered as ‘contaminants’ of EV preparations [25,26]. Concerning nucleic acids, distinct RNA classes have been found in EVs including functional messenger RNA (mRNA), non-coding RNA (microRNAs (miRNAs), long non-coding RNAs, and circular RNAs) and other regulatory RNA molecules (transfer RNA, small nucleolar RNA, mitochondrial RNA, vault RNA, among others). *Valadi* et al. were the first to report the existence of mRNAs and miRNAs in EVs and their functional transfer to EV-targeted cells [27]. It has been proposed that cells may use RNA-binding proteins with distinct binding preferences to secrete miRNAs in exosomes [28] or, even, discard unnecessary or inhibitory RNAs. Not only the type of nucleic acid species but also the quantity is crucial for functional impact. Despite large amounts of data over the last years supporting the role of nucleic-acid-associated EVs on cell communication, the specific sorting, transport, and effect of these RNA molecules on EVs is still a poorly understood process that deserves further attention. In this regard, non-vesicular genetic material [29] and EVs could be co-isolated depending on the vesicle isolation procedures, not reflecting true EV-released contents. Moreover, isolation methods influence the yield of recovery of EV-derived RNA [30]. Thus, discrimination of such confounders and reassessment and integration of data is needed prior to assigning any function to EV-associated nucleic acids.

Another layer of complexity in this constantly evolving field includes technical challenges inherent of EVs. The biological, biochemical, and biophysical properties of EVs often overlap between distinct EV subtypes and might be cell-type- and cell-region-specific hampering their separation, discrimination, and analysis [31]. Moreover, preanalytical basic considerations (sample source, phlebotomy procedure, anticoagulant, temperature, storage, etc.) are of utmost importance to avoid contamination of cell-derived vesicles with other non-EV structures and protein aggregates, among others. Equally vital is the correct reporting of methods to ensure reproducibility and standardization. Thus, isolation methods of choice for EV studies (used in combination or even modified) should depend on yield, purity, integrity, concentration, downstream applications, and scientific question, following the recommendations by the Minimal information for studies of extracellular vesicles guidelines ISEV position paper [15]. A methodological consensus framework for the identification and characterization of EVs in cardiovascular medicine studies has very recently been established [32]. While all these obstacles have to be overcome yet, tremendous advances in EV research simultaneously to the emergence of new technologies will undoubtedly reshape our approach to studying extracellular vesicles.

## 3. EVs as Pathophysiological Drivers of Atherothrombosis

EVs orchestrate the entire spectrum of atherosclerotic disease phases by means of their intercellular and extracellular communication and adhesion between blood and vessel wall [33]. Human atherosclerotic plaques, from initial lesions to advanced stages, contain EVs expressing markers from vascular and blood cells (leukocytes, red blood cells (RBCs), smooth muscle cells, and endothelial cells (ECs)) [34,35,36]. Therefore, it seems plausible that EVs from most cell types of the cardiovascular system participate in the atherosclerotic process. It should be taken into consideration that isolation of EVs from tissues (by homogenization, dissociation, or disruption) preserving their intact biochemical properties is still a challenging procedure that should be further developed. Nevertheless, the landmark study from Leroyer et al. [35] reported that plaque EVs derive mainly from white blood cells (WBCs), demonstrating the existence of an inflammatory milieu. Of interest, plaque EVs favored the balance towards a proinflammatory environment in the culprit lesion [37] and induced T-cell proliferation in a dose-dependent manner, contributing to vascular inflammation and plaque development [36]. A growing body of evidence supports the notion that immuno-inflammation plays a role in the pathogenesis of atherothrombotic disease either by promoting the disease or lesion resolution and vascular repair. Here, we will unravel the immuno-modulatory effect of EVs in the different stages of atherothrombotic disease.

### 3.1. Atherogenesis

Endothelial dysfunction (ED) characterized by a proatherogenic environment is an early sign of atherosclerosis development [38]. Under pathological conditions, such as risk factors or injury, circulating EVs contribute towards a dysfunctional endothelium [39,40,41,42,43,44,45,46,47,48,49,50]. Beyond the well-studied traditional cardiovascular risk factors, atherothrombosis is spearheaded by multiple risk factors such as clonal hematopoiesis and air pollution [51]. It has recently become apparent that particulate matter (PM) could induce an inflammatory response and EC damage favoring the initial formation of atherosclerotic lesion. EVs derived from PM-exposed alveolar epithelial cells fueled inflammatory signaling via inhibitor of the nuclear factor kappa alpha (IκBα)–nuclear factor kappa-light-chain-enhancer of activated B cells (NF-κB)–vascular cell adhesion protein 1 (VCAM1) axis [52]. Nevertheless, EVs shed after PM exposure could also mediate crosstalk between EC-immune cells to cope with the inflammatory assault [53], a response impaired in overweight subjects at increased cardiovascular risk [54].

EVs of several cellular origins (mainly derived from ECs) upon distinct inflammatory stimuli (oxidized low-density lipoprotein (LDL), angiotensin II, hypertension, visceral adipose tissue, and infection) or isolated from diseased patients (circulating EVs) directly cause harmful effects on physiological EC function and vasorelaxation by impairing nitric oxide (NO) bioavailability via regulation of NO, nitric oxide synthase, nicotinamide adenine dinucleotide phosphate (NAPDH) oxidase, and prostacyclin as well as reactive oxygen species (ROS) and endoplasmic reticulum stress [55,56,57,58,59,60,61,62]. RBCs play key roles in vascular homeostasis. Not only altered RBC function but also RBC-derived EVs could reduce NO bioactivity and induce ED [63,64]. Likewise, excessive erythrocytosis exhibited by Andean highlanders also induce ED by means of dysfunctional circulating EVs [65]. Extracellular signal-regulated kinase (ERK) 1 embedded in platelet EVs from a mouse model of diabetes induce ED via intracellular adhesion molecule 1 (ICAM-1) [66]. Senescent EC-derived MVs (eMVs) from ACS patients induce premature ED and thrombogenicity through the angiotensin II–induced NADPH oxidase system [67]. On top of this detrimental vascular effect, EVs are also responsible for endothelial permeability [68,69]. Several works suggest that the disturbance of EC barrier function could also be mediated through apoptosis by EVs [70,71,72,73], whereas other studies ascribe to them a protective role as a way of reducing intracellular levels of caspase-3 [74]. Along the same line, Jansen et al. showed that eMV uptake in ECs via annexin I/PS receptor-p38 signaling protects against apoptosis [75]. Li et al. have demonstrated that endothelial progenitor cell (EPC)-derived EVs inhibit ferroptosis and alleviate atherosclerosis vascular injury via the miR-199a-3p/specificity protein 1 axis [76]. Of interest, EVs in basal conditions or from healthy subjects do not show deleterious effects to the vascular wall [77,78], highlighting that the impact of EVs in vivo is cell source- and context-dependent [79,80,81,82,83].

### 3.2. Atheroinflammation

An additional key feature of early atherosclerosis is the activation of the endothelium and, subsequently, the adhesion and recruitment of immune cells to the vasculature [84]. Importantly, EVs contribute in a multifaceted fashion to instigate vascular inflammation by increasing levels of adhesion molecules, ROS, and proinflammatory cytokines (e.g., interleukin (IL)-6 and -8) [85,86,87,88]. Specifically, platelet-derived EVs (pEVs) have shown in vitro to enhance the expression of adhesion molecules including E-selectin, ICAM-1, and VCAM-1 [89,90]; thus, favoring the adhesion of monocytes to the inflamed endothelium. Of note, P-selectin-enriched pEVs were found to promote leukocyte–leukocyte interactions leading to monocyte diapedesis through the endothelium even when flow conditions were not favorable [91]. Such chemo-attraction can be regulated by multiple mechanisms such as the transfer of Regulated on Activation, Normal T cell Expressed and Secreted (RANTES) protein to ECs [92] and might contribute to long-term leukocyte differentiation [93]. In this regard, apoptotic-platelet-derived EVs can mediate the polarization towards M2 monocyte and resident macrophages. pEVs not only facilitate leukocyte adherence to the arterial wall but also to the atherosclerotic plaque [94]. In contrast, pEVs can also reshape the secretome of angiogenic early outgrowth cells (EOC) magnifying EOC-driven endothelial regeneration and promoting vascular protection [95]. Latter findings emphasize the importance of the environment in the final EV-directed effect.

A similar effect of chemo-attraction and transmigration of leukocytes to ECs towards the plaque is induced by EVs from other cellular sources [96]. Of note, neutrophil-derived EVs (nEVs) cause myeloperoxidase-mediated damage of vascular ECs [97]. nEVs shuttle Cox-1 substrate arachidonic acid (AA) from neutrophils to platelets, thereby fostering thromboxane A2, EC activation, and neutrophil recruitment in an experimental model of pulmonary hypertension [98]. Thus, nEVs are involved in platelet–neutrophil crosstalk. Leukocyte-derived EVs released upon proinflammatory triggers can adhere to ECs and promote further leukocyte adhesion in a positive feedback loop [99,100,101]. One of the mechanisms by which EVs exert their proinflammatory activity is by harboring mitochondria or mitochondria-derived proteins, the so-called mitovesicles [102,103]. Extracellular mitochondria cause inflammatory responses to injury because they bear damage-associated molecular patterns [104,105], mitochondrial DNA [106], and oxidized proteins [107]. For instance, monocyte-derived mitovesicles can induce type I interferon and tumor necrosis factor (TNF)α response in ECs [108]. It has also been described that coronary artery disease (CAD) patients with low levels of mitochondrial oxidase subunit I in monocyte-derived EVs are at higher risk for new cardiovascular events [109]. pEVs released from thrombin-stimulated platelets also have embedded mitochondria that act as a substrate for phospholipase A2 IIA [110]. Altogether, EVs selectively enriched with mitochondrial material promote the expression of inflammatory mediators and drive leukocyte recruitment in the vasculature. Another mechanism involved in the vascular pro-inflammatory milieu is the exporting and transferring of miRNAs to target cells. Endothelial-derived EVs (eEVs) have shown to transport miRNA-155 and reprogram monocytes into an M1 proinflammatory phenotype [111] and miRNA-92a to macrophages promoting an atheroprone phenotype [112]. Not only miRNAs but also post-translational modifications can influence EV-driven inflammatory signals to ECs. Recently, Yang et al. have shown that monocyte–EC interactions responsible for EC inflammatory phenotype depend on EV-associated glucose transporter 1 and glycosylation [113].

Conversely, EVs can also act as inflammatory repressors. Endothelial-derived EVs bearing the endothelial protein C receptor-activated protein C complex induce protease-activated receptor (PAR) 1-dependent survivable, cytoprotective, and anti-inflammatory effects on ECs [114]. MVs derived from lipid-loaded macrophages (CD16^+^) and containing LRP5 induce anti-inflammatory phenotypes in macrophages in an autocrine and paracrine fashion [115]. The EC-derived miRNA-143/-145 cluster is delivered to adjacent smooth muscle cells, through a MVs-mediated mechanism, to induce a vasculoprotective phenotype [116]. Neutrophil-derived EVs induce the release of transforming growth factor (TGF) β1 from macrophages with anti-inflammatory effects [117] that could be reinforced by the expression of annexin I on the surface of nEVs [118]. Moreover, ICAM-1 is downregulated by miRNA-222 embedded in eEVs [119]. Another example of a favorable vesicle-mediated miRNA transfer is the anti-inflammatory miRNA-10 targeting monocyte NF-κB pathway [120]. When EVs are taken up by B cells and monocytes, they are able to reprogram target cells towards an anti-inflammatory profile dependent on distinct factors [121]. Continuing with this dichotomy of pro- and anti-inflammatory properties of EVs and in an attempt to untangle the particular contribution of each vesicle subtype to the early phase of atherosclerotic disease, Hosseinkhani et al. provided evidence of a differential immunomodulatory content (inflammatory signals, chemokines, and cytokines) between small and large EVs released from activated ECs [122,123]. Specifically, sEV stimulate monocyte migration while lEVs are prompt to induce ICAM-1 expression and inflammation profile in target cells.

Even though most of the data regarding EVs as pro- and anti-inflammatory mediators in atherosclerosis derives from in vitro models, several studies with EVs from pathological conditions have been conducted [124,125,126,127,128]. Deepening to the mechanism, monocyte-derived EVs carrying IL-1β are involved in EC activation [101] and, consistently, IL-1β-rich eEVs in inflammation in the setting of acute coronary syndrome (ACS) [129]. Remarkably, MVs within atherosclerotic plaques are able to recruit pro-inflammatory cells through transfer of ICAM-1 to ECs [130].

In addition to chronic vascular inflammation, atherosclerosis is also characterized by an impaired resolution response leading to non-resolving inflammation persistence. EVs circulating in plasma could also exert such immunomodulatory potential in atherosclerosis. For instance, deregulated anti-inflammatory and pro-resolving molecules (CD5L and Resolvin E1 bioactive lipid mediator) in circulating EVs contribute to the systemic hyperinflammation in the context of chronic liver disease [131]. In atherosclerotic inflammation, miR-155 was found decreased in a model of atherosclerosis regression and increased in urinary EVs (uEVs) from unstable CAD patients. Interestingly, CAD progression was associated to increased CD45^+^ and CD11b^+^ uEVs, and decreased in CD16^+^ uEVs, pointing at miR-155 and uEVs as a potential prognostic marker of disease progression and severity and a therapeutic target [132].

### 3.3. Lesion Progression

#### 3.3.1. Atherogenic Foam Cell Formation

The adaptive immune system appears at the center of human ASCVD development. Oxidized (ox-LDL) and aggregated LDL particles are taken up by the macrophages that, by removing the excess of lipids, become foam cells. As a consequence, macrophage lipid accumulation via shedding of EVs (1) inhibits their clearance [133], (2) leads to the formation of oxidation-specific epitopes in a subset of cholesterol-induced EVs [134], and (3) amplifies the inflammatory response. For instance, monocyte-derived EVs promote T-cell infiltration in atherosclerotic plaques [135]. More specifically, macrophages have shown to promote proinflammatory and proatherogenic phenotypes in recipient cells through secretion of EVs conveying miRNAs. Upon lipid uptake, atherogenic (cholesterol-loaded) macrophages secrete EVs and deliver miRNA-146a to naïve macrophages where it represses the expression of specific promigratory target genes (insulin-like growth factor 2 mRNA-binding protein 1 and human antigen R), leading to decreased macrophage migration and potential entrapment in the atherosclerotic plaque [133]. Finally, MV-mediated miR-223 transfer reinforces the macrophage activation loop [136]. Ox-LDL also induces EV release from ECs containing miR-155 which promotes M1 macrophage polarization [111]. Indeed, beyond macrophages and foam cells, ECs and platelets also secrete EVs that regulate macrophage polarization and contribute to atherosclerotic plaque progression. TNFα or low shear stress-induced EC-derived EVs also target macrophages [137]. Activated pEVs stimulate ox-LDL phagocytosis and inflammatory cytokine release by macrophages [138]. Thereafter, macrophages and foam cells undergo apoptosis, either by their phagocytosis of ceramide and AA-rich EVs [70,139] or by the EV-driven transfer of programmed-cell-death-related caspases [140] even in SMCs [73]. Lymphocyte accumulation in inflamed arteries can also display a regulatory and atheroprotective function. B-cell-derived natural IgM recognizes EVs bearing oxidation-specific epitopes and represses their inflammatory signaling and pathological endeavors [141]. Moreover, natural IgM antibodies inhibit MV-driven coagulation and thrombosis [142].

#### 3.3.2. Vascular Smooth Muscle Cell Proliferation, Migration, and Phenotype Switching

During lesion growth, vascular smooth muscle cells (VSMC) located in the media change from a contractile to a proliferative phenotype and migrate into the intima layer. VSMC cell proliferation and migration, another key step in the development of atherosclerosis, is also influenced by EVs either from platelets [143] or foam cells [144]. Platelet-released EVs induce a pro-inflammatory phenotype in VSMC affecting vascular remodeling [143]. In addition, platelet-derived MVs have shown to promote VSMC proliferation and migration via a platelet-derived growth-factor-independent mechanism [145,146] and calcium oscillations and transient receptor potential vanilloid 4 [147], respectively. Macrophages release EVs rich in cholesterol that can be taken up by VSMC [148]. The contribution of macrophage foam cell-derived EVs to enhance VSMC adhesion and migration was shown by the transfer of integrins and subsequent downstream activation of ERK and Ak strain transforming (AKT) [144] and the transport of miR-19b-3p and targeting juxtaposed with another zinc finger gene 1 [149]. Those EVs rich in tissue factor (TF) have the capacity to modulate VSMC migration though PAR interaction [150]. Another study found that VSMC-derived EVs intervene in autocrine VSMC proliferation by upregulation of mitogen-associated protein kinase [151]. Interestingly, other studies address this question with a translational perspective. EVs bearing Ras-related protein 1 from patients with metabolic syndrome favored VSMC migration and proliferation [152]. Plasma EV containing miRNA-501-5p promotes VSMC phenotypic modulation through targeting Suppressor of mothers against decapentaplegic homolog (Smad) 3 in patients with in-stent restenosis one year after coronary stent implantation [153]. Conversely, several studies emphasize the fact that EVs and their miRNA content present an atheroprotective effect on VSMCs. In this regard, miRNA-223-EVs inhibited VSMC migration and proliferation, thereby decreasing plaque size [154]. Similarly, both high shear stress and Krüppel-like factor 2-expressing ECs-derived EVs enriched in the miR-143/145 cluster prevented VSMC dedifferentiation [116]. Moreover, macrophage-derived EVs also transmit miR-503-5p into VSMCs and inhibit their proliferation and migration via the Smad7-Transforming growth factor-β axis [155]. Finally, MVs reduced VSMC proliferation, migration and subsequent neointima formation by delivering functional miR-126 into recipient VSMCs [155]. Even EVs from adipose mesenchymal stem cells were involved in the regulation of VSMC phenotype to limit intimal hyperplasia [156].

#### 3.3.3. Intravascular Calcification

Under pathological stimuli, EVs released by VSMCs and macrophages aggregate between collagen fibers and serve as a platform for ectopic mineralization eliciting micro- and macrocalcifications in the vessel wall. Vascular calcification in atherosclerosis shows a bimodal behavior in terms of risk and impact; while it has been shown that microcalcifications have a burdening effect on plaque rupture due to the low number of inflammatory cells and being more prone to rupture, higher amounts of calcification within stable plaques at various phases of atherosclerosis progression have also been found. Cellular-derived EVs from VSMCs, ECs, and platelets indisputably regulate the loss of the VSMC contractile phenotype and impact vascular calcification [157,158,159] favoring plaque calcification and atherogenesis [160,161,162,163]. EVs from dysfunctional endothelium [164] and senescent ECs [158,165] promote vascular calcification. VSMCs also release calcifying EVs themselves [166,167]. One described mechanism driving the VSMCs towards calcification involves the regulation of calcifying EVs by sortilin [166]. Oxidative stress also intervenes in this process. VSMC phenotype switching caused an increased Nox5 expression that was responsible for the subsequent extracellular calcium entry, ROS production, reduced contractile phenotype, and enhanced EV release fostering VSMC calcification [168].

#### 3.3.4. Necrotic Core

EV-mediated cell death [73,139] triggers necrotic core formation and, in turn, lipid-rich necrotic core contains high amounts of thrombogenic EVs as well [35,36,169], conferring a procoagulant potential and instability to the atherosclerotic lesion and shifting towards a vulnerable plaque.

### 3.4. Advanced Lesion and Plaque Rupture

Atherosclerotic plaque erosion or rupture and the subsequent thrombosis leads to, eventually, acute ischemic syndromes. The interdependence between innate immune cells and platelets is essential for plaque destabilization and thrombotic occlusion [170,171].

#### 3.4.1. Arterial Neoangiogenesis and Intraplaque Hemorrhage

During the last steps of atherosclerosis development, an increased number of vasa vasorum brings about intraplaque hemorrhage, a highly inflammatory environment, and a rapid activation of the coagulation cascade and platelet aggregation forming a fibrin mesh [172,173]. Several studies support the notion that EVs exert regulatory roles in these events [174,175]. EPC-EVs activate ECs and stimulate angiogenesis [176]. Inflammation-induced EC-derived EVs enhanced vascular endothelial growth factor B expression in pericytes, which in turn might mediate pathological neovascularization and vascular leakage in response to an inflamed endothelium [177]. Atherosclerotic EC-released EVs promoted angiogenic responses in ECs by a thrombospondin-1-depedent mechanism [112]. Communication between ECs and immune cells plays key roles in disease development. Spleen EC-derived VCAM1^+^- EVs mobilize splenic monocytes in myocardial infarction (MI) [178]. Our group has reported that hypoxic ECs release endothelial-cell-derived MVs rich in TF and transport miRNA-126 that induces monocyte recruitment into the ischemic zone, reprogramming of monocytes and their differentiation into EC-like cells, and angiogenic response, which altogether improve tissue reperfusion [179,180]. Of note, TF-containing eMVs could interact via paracrine signaling with other microvascular ECs and trigger angiogenesis ex vivo and postischemic collateral vessel growth in vivo [181]. MVs from human atherosclerotic plaques promoted in vivo angiogenesis [182]. Of interest, in vitro studies showed that the mechanism was mediated by MVs expressing the CD40 ligand [182]. Similarly, MVs from ischemic muscle induced postnatal vasculogenesis [183]. Fibrinolytic activity of EC and WBC-derived EVs could also favor angiogenesis and hamper thrombus dissolution [184]. Furthermore, Loyer et al. found that small and large cardiomyocyte-derived EVs are locally released from the heart to regulate the inflammatory process in a mouse model of myocardial ischemia [185]. Indeed, ischemia-induced cardiosomes stimulate cardiac angiogenesis both in vitro and in vivo [186]. Besides intraplaque neovascularization and bleeding, plaque, EC, and immune-cell-derived EVs influence endothelium denudation and fibrous cap integrity. EVs exert structural extracellular matrix degradation and fibrous cap weakening via metalloprotease activities [37,187,188,189,190,191,192,193]).

#### 3.4.2. Thrombus Formation after Rupture

Fibrous cap disruption within the atherosclerotic plaque results in the exposure of subendothelial extracellular matrix (collagen and von Willebrand factor) and thrombogenic substances and, thus, a huge release of tissue factor triggering platelet activation, aggregation, and thrombus formation in the damaged area [194]. EVs from platelet/monocyte aggregates are capable of modulating human atherosclerotic plaque reactivity [195]. Under in vitro simulated conditions of coagulopathy, EV from different cellular origins (EC and platelet) and at distinct concentrations showed a divergent but procoagulant effect within the coagulation process [196]. Numerous studies have dissected in vitro and in vivo the procoagulant effect of EVs, mainly MVs, leading to thrombosis [142,197,198,199,200,201,202,203,204,205,206,207]. Procoagulant EVs within the necrotic core of atherosclerotic plaques provoke the burst of the coagulation cascade upon plaque rupture [169] being TF^+^-MVs the main initiators [208,209,210]. TF is functionally transferred to monocytes through MVs [211] in a PAR2-filamin A-mediated process [212]. The fact that the proinflammatory cytokine IL-33 [213] and EC-derived EVs [214] trigger the release of procoagulant TF^+^-MVs underscores the synergy between inflammation and thrombosis. TF^+^-MVs are also engulfed by platelets and trigger platelet aggregation [215,216]. In addition, TF-rich monocyte-derived EVs are recruited at the thrombus site [217]. TF shares the limelight of plaque thrombogenicity with phosphatidylserine. Negatively charged PS exposure in EV surface confers procoagulant activity [218], enables the concentration of factors VII/VIIa in the EV membrane [219], enhances platelet adhesion to the endothelium, and it is important for TF function, thrombin generation, and clot formation [220]. Beyond these two key factors (TF and PS), EVs were shown to directly modulate the clotting. A seminal study on leukocyte-derived EVs expressing P-selectin glycoprotein ligand-1 (PSGL-1) on their surface deconvolutes the crosstalk between platelets and TF-rich immune cells at the lesion site. Interactions between platelet adhesion molecule P-selectin and PSGL-1^+^-EVs allowed higher recruitment and activity of TF perpetuating thrombus formation [221]. Not only WBC-derived EVs participate in this dynamic process, circulating and platelet-derived MVs also enhance thrombus formation and growth on thromboactive substrates [205]. We also found that pEVs bearing surface epitopes of platelet adhesion and activation in perfused blood tend to bind more to adhered platelets and prothrombotic surfaces stimulating further platelet deposition and thrombus growth [207]. Interaction of platelets with subendothelial matrix, immobilized platelet surfaces [222], or fibrin [223] depends on pEV-associated αIIbβ3a integrin [224].

Several other platelet biological components contribute to this phenomenon such as protein disulphide isomerase [225], factors VIII and Va [226,227], and bioactive lipids [228]. Platelet-derived EVs also modulate EC–monocyte interactions [89] likely inducing enhanced expression of cell adhesion molecules under high shear stress conditions [90]. Interestingly, pEVs support megakaryocyte differentiation and platelet production that is relevant to keep a physiological level of circulating platelets after their consumption during thrombosis [229]. Recently, we have investigated the proteostatic characteristics of EVs shed from platelets upon thrombin activation by an untargeted proteomic approach [230]. We have identified proteins involved in cell–cell interaction and signaling events underlying thrombus formation such as CUB domain-containing protein-1 or membrane glycoprotein gp140, fermitin family homolog 3 or kindlin-3, and the novel pEV-associated protein protocadherin-α4, among others. Deciphering platelet and cell signaling and biology by proteomics technique [230,231] will help to unveil paracrine regulation of EVs and provide new therapeutic targets involved in occlusive thrombus formation and atherothrombosis.

## 4. Extracellular Vesicles as Diagnostic and Prognostic Biomarkers

EVs are released in healthy physiological steady conditions. Clinical ASCVD development is associated with high release of EVs. Assessment of vascular status is crucial for atherosclerosis prevention and to this aim EVs can serve as potential biomarkers in the clinics [38,232]. Molecular footprints of EVs provide clues about their cellular origin under healthy and diseased states, configuring a precious biomedical tool for liquid biopsy, easily detectable in body fluids. Changes in circulating EVs of different cellular origin have been widely reported for almost all cardiovascular risk factors [233,234,235,236] and pathologies [237,238,239] reflecting their pathophysiological severity. Beyond diagnostic use, EVs have been suggested for the prediction of major adverse cardiovascular events (MACE) [230,231,232,233,234,235,236,237,238,239,240,241,242,243] and mortality [244]. Circulating procoagulant MVs were found at higher levels in patients with ACS than healthy subjects [238,239,240,245,246,247,248,249,250,251,252,253,254,255]. Table 1 lists representative studies showing EV changes in cohorts of patients with distinct ASCVD risk factors and disorders with potential utility as diagnostic and prognostic biomarkers.

Among different cell-derived EVs, EVs from endothelium stand out due to their capacity to mirror ED [256,257], correlate to cardiovascular outcomes [258,259], and predict disease severity [260]. Circulating EVs associate to ED and plaque rupture in ACS patients [261]. Associations between circulating EVs and angiographic lesions have also been reported [262,263]. Circulating EV levels correlated with a higher calculated 10-year Framingham CAD risk [264]. Risk assessment is critical in primary prevention of ASCVD, especially for asymptomatic patients with subclinical atherosclerosis. In this context, EVs emerge as promising biomarkers for CV risk stratification. We found that patients with familial hypercholesterolemia (FH) presenting subclinical lipid-rich atherosclerotic plaques have significantly higher amounts of circulating lymphocyte-derived (CD3^+^/CD45^+^) EVs than FH patients with fibrous plaques [50] in agreement with other studies that reported high levels of EVs from T lymphocytes in subjects with essential hypertension [235] and about to develop a MACE [265]. Thus, lymphocyte-derived EVs might help to map atherosclerotic plaque burden. Accordingly, increased circulating levels of leukocyte-derived EVs in patients with unstable atherosclerotic plaques signal for plaque vulnerability [246]. Furthermore, we also found that asymptomatic high cardiovascular risk FH patients have significantly higher number of circulating prothrombotic platelet EVs [266]. Patients with FH, despite being treated according to guidelines, have ongoing innate immune cell and platelet activation. Indeed, in asymptomatic high cardiovascular risk FH patients from the same cohort, pan-leukocyte-derived and activated neutrophil-derived circulating EVs as well as EVs bearing markers of platelet activation were significantly increased in patients about to suffer an ischemic event [242]. This specific EV signature is a prognostic marker able to improve the prediction of clinical events if included in the risk factor models. Altogether, it is clear that multiple biomarker-based strategies for patient stratification including EVs hold promise in precision medicine.

**Table 1:** Extracellular vesicles (EVs) as potential markers of ASCVD diagnosis and prognosis. Studies showing EV changes in distinct ASCVD risk factors and pathologies.

Clinical Condition/Context	EV Subtype	Cargo	Reference
Cardiovascular risk factors	↑ eEV (CD144^+^, CD105^+^) (*Hypertension*)		[267]
	↑ lEV (CD3^+^) (*Hypertension*)		[235]
	↑ eEV (CD31^+^/42^−^; CD31^+^/AnnV^+^), pEV (CD31^+^/42^+^) (*Diabetes*)	miR-126	[236]
	↑ lEV (CD45^+^), nEV (CD15^+^), pEV (CD62P^+^) (*Familial hypercholesterolemia [FH]*)		[242]
	↑ pEV (CD42a^+^, CD62P^+^) (*Obesity*)		[268]
	↑ pEV (CD41^+^), eEV (CD62E^+^), lEV (CD45^+^) (*Smoking*)	CD40L	[269]
Pulmonary hypertension	↑ eEV (CD31^+^, CD144^+^, CD62E^+^), lEV (CD45^+^)		[260]
Endothelial dysfunction	eEV (CD31^+^/CD42b^−^)		[255]
	eEV (CD31^+^/AnnV^+^)		[256]
FH and subclinical atherosclerosis	↑ lEV (CD3^+^/CD45^+^)		[50]
	↑ pEV (TSP1^+^/CD142^+^)	Tissue factor	[266]
Coronary artery disease (CAD)	↑ CD31^+^/Annexin V^+^ EV		[258]
	↑ eEV (CD31^+^), pEV (CD42b^+^)	miR-126, miR-199a	[241]
	↑ eEV (CD31^+^/AnnV^+^)		[256]
	↑ eEV (CD31^+^ and CD51^+^)		[238]
	↑ eEV (CD31^+^)		[270]
Arterial hypertension and coronary artery disease	↑ eEV (CD31^+^/41^−^, CD62E^+^, CD144^+^)		[271]
Diabetes and CAD	↑ eEV (CD62E^+^, CD31^+^)	↓LAP(TGF-β1), PD-ECGF, PF4, TSP1	[233]
Coronary heart disease	↑ eEV (CD31^+^/CD42^−^, CD144^+^)		[272]
	↑ pEV (CD41^+^), eEV (CD144^+^)		[273]
	↑ eEV (CD31^+^ and CD146^+^)		[248]
Stable angina	↑ eEV (CD31^+^), pEV (CD41^+^)		[274]
Acute coronary syndrome	↑ pEV (CD31^+^, CD41a^+^)		[275]
	↑ eEV (CD146^+^)		[276]
	↑ eEV (CD144^+^), ErEV (CD235a^+^), pEV (CD41a^+^)		[261]
Myocardial infarction (MI)	↑ CM-EV and eEV (CD31^+^/CD41^−^)	Caveolin-3, TnT	[185]
	↑ ErEV (CD235a^+^)		[207,252]
	↑ eEV (CD144+)		[277]
	↑ l/mEV, eEV, ErEV, activated vascular cell-EV, (CD66b^+^/62E^+^/142^+^ for coronary thrombosis)		[245]
	↑ eEV (CD154^+^, CD62E^+^), pEV (CD62P^+^)		[278]
	↑ eEV (CD31^+^), pEV (GPIbα)		[247]
	↑ eEV (CD31^+^, CD146^+^), pEV (CD42b^+^)		[254]
	↑ pEV (CD61^+^), mEV (CD14^+^)	Tissue factor	[244]
	↑ lEV(CD45^+^), pEV (CD61/62P^+^), ErEV (CD235a^+^)	Lactadherin, Fbn	[279]
	↑ eEV (CD144^+^), pEV (CD62P^+^/41), mEV (CD14^+^)	Tissue factor	[280]
MI and stable angina	↑ pEV (CD51^+^/61^+^), eEV (CD42^−^/31^+^), mEV (CD14^+^)	Tissue factor	[250]
MI in chronic kidney disease	↑ eEV (CD154^+^, CD62E^+^), pEV (CD62P^+^)		[278]
Ischemic cerebrovascular disease	↑ eEV (CD144^+^, CD31^+^, CD62E^+^/41a^−^, AnnV^+^)		[281]
	↑ eEV (CD62E^+^, CD31^+^/, CD42b^−^ and Annexin V^+^)		[237]
Stroke	↑ NPC-EV (CD34^+^, CD56^+^)		[282]
	↑ pEV (CD41^+^)		[283]
	↑ eEV (CD62E^+^)		[259]
	↑ eEV (CD31^+^, CD144^+^, CD146^+^), lEV (CD45^+^)		[243]
Peripheral arterial disease	↑ pEV (CD41^+^/61^+^)	Calprotectin	[284]
	↑ cEV	Sonic hedgehog	[285]
	↑ eEV (CD144^+^)		[286]
Venous thromboembolism	↑ eEV (CD62E^+^), mEV (CD14^+^)	PSGL-1^+^	[287]
Atrial fibrillation and stroke	↑ eEV (CD41^−^/31^+^), pEV (CD61^+^)		[288]
Autoimmune chronic inflammatory diseases	↑ eEV and pEV		[289]

AnnV indicates annexin V; cEV, circulating extracellular vesicle (EV); CD, cluster of differentiation; CM-EV, cardiomyocyte-derived EVs; eEV, endothelial-derived EV; ErEV, erythrocyte-derived EV; Fbn, fibrinogen; LAP(TGF-β1), latency-associated peptide (transforming growth factor β1); lEV, leucocyte derived EV; mEV, monocyte-derived EV; nEV, neutrophil-derived EV; NPC-EV, neural precursor cells; PD-ECGF, platelet-derived endothelial cell growth factor; PF4, platelet factor 4; pEV, platelet-derived EV; PSGL-1, P-selectin glycoprotein ligand-1-positive; TnT, troponin T; and TSP1, thrombospondin 1; ↑, increase.

When studying circulating EVs as markers of disease it is relevant to consider confounders. We know that EV levels can be impacted by sex [290], age [291], pregnancy [292], a high-fat meal [293], and exercise intensity [294,295,296]. Indeed, skeletal muscle myofibers are a major source of EVs, more than white adipose tissue, that can reach the circulation in vivo [297]. Large clinical trials to fully demonstrate EV potential are an indispensable pre-requisite before entering the clinical arena.

### Pharmacological Modulation of Extracellular Vesicles

EVs are amenable to pharmacological intervention. Several drugs have shown the ability to influence EV shedding as well as EV composition. For instance, antioxidants [298], antihypertensive drugs (angiotensin II receptor antagonists [299] and calcium channel blockers [300]), lipid-lowering therapy (statins [301] and ezetimibe [302]), and antiplatelet drugs (acetylsalicylic acid [303], GPIIb/IIIa inhibitors [304], clopidogrel [305], and ticlopidine [306]) have shown to reduce EV levels, as reviewed elsewhere [4]. Recently, new antidiabetic drugs have been studied in this regard. Circulating EVs from CAD patients via the angiotensin signaling induce higher expression of sodium-glucose cotransporter (SGLT)-2 protein promoting endothelial senescence and dysfunction as well as pro-atherothrombotic responses in coronary ECs; effects that can be targeted by SGLT-2 inhibitors [307]. In a similar fashion, thoracic adipose tissue secretes ceramide 16:0 metabolite within EVs that enhance vascular redox state in the obesity setting, a phenomenon that is amenable to treatment with glucagon-like peptide 1 receptor antagonist liraglutide [308]. Liraglutide ameliorated pro-atherogenic eMV release in women with gestational diabetes, highlighting atheroprotective effects of liraglutide [309]. Importantly, the anti-inflammatory drug colchicine, which has demonstrated beneficial effects on MACE after MI [310] and in patients with chronic coronary disease [311], inhibits NLR family pyrin domain containing 3 (NLRP3) inflammasome and reduces EV-associated NLRP3 protein levels [312].

## 5. Extracellular Vesicles as Therapeutic Tools

**Figure 1:**
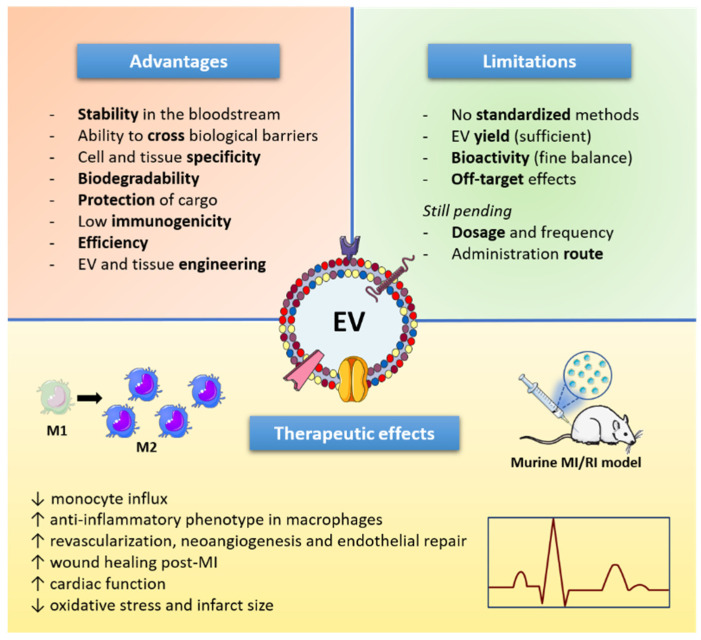
Schematic summary of extracellular vesicles (EVs) as therapeutic tools. EV indicates extracellular vesicle; MI, myocardial infarction; ↑, increase; ↓ decrease.

EVs are considered as transfer systems capable of delivering biological cargoes in health and disease (Figure 1). Given their beneficial effects on vascular homeostasis and atheroprotection and their recognition as the next generation of cell-derived therapeutics [313], EVs show promising perspectives for ASCVD therapy as has recently been suggested for pEVs [314]. EVs offer multiple advantages as drug delivery vehicles such as stability in the bloodstream, biodegradability, ability to cross biological barriers and target specific cells or tissues, protection to their loaded content, low immunogenicity (if the same patient’s sample is used), among others [315]. In cardiovascular medicine applications, we could add the fact that their biodistribution as intravenously-injected EVs could reach the whole vascular network. As major impediments and due to multiple functional properties displayed by EVs (both beneficial and detrimental), tight control of their ultimate mission as well as cell and tissue specificity should be stably achieved to avoid off-target effects [316].

Up to now, one of the areas where the therapeutic potential of EVs has raised interest and has been widely explored is in cardiac regenerative therapies. EVs from distinct cell types have shown to impact on cardiac cells providing a tissue-reparative phenotype. pEVs induced VEGF-dependent revascularization after chronic cardiac ischemia [317]. Concerning EVs from heart immune cells, dendritic-cell-derived EVs infiltrate the ischemic myocardium and act as activators of CD4^+^-T-cells and improve wound healing post-MI [318]. Macrophages-derived EVs enhance inflammation in injured cardiac cells [319]. Inflammation-induced EC-derived EVs regulated the expression of angiogenic genes in pericytes [320], indicating the crosstalk between cardiac cells and pericytes in myocardial remodeling. EVs have recently gained attention in therapeutics as paracrine contributors to reparative functions of stem cells (mainly from endothelial progenitor or mesenchymal (MSC), cardiac, embryonic, and induced pluripotent stem cells) in the heart. The vesicular fraction of conditioned media from hypoxic MSC was able to reduce infarct size and oxidative stress and improve cardiac function, thereby potentiating myocardial viability and preventing further damage to the heart after MI [321,322]. In addition, stem- and progenitor-derived EVs injected intramyocardially and intravenously in preclinical murine models of myocardial ischemia/reperfusion injury, were able to improve cardiac function by modulating monocyte and macrophage activity and their polarization into anti-inflammatory [323].

miRNA-transferring EVs have also recently been considered as therapeutic vectors following myocardial ischemia [324]. miRNA-126 MVs stimulated re-endothelization following vascular injury by targeting Sprouty-related EVH1 Domain Containing-1 protein [325]. Upon the inflammatory insult, ECs release VCAM-1^+^–EVs that promote splenic monocyte mobilization and transcription activation in acute MI [178]. Therapeutic targeting of the EV-associated VCAM-1 axis after AMI may specifically limit splenic monocyte cell movement and modulate the inflammatory phase of heart injury, thereby improving cardiac function and long-term prognosis. In fact, therapeutic immunomodulation of the neutrophil response to MI using EV vectors (EC-derived EVs containing VCAM1 and miR-126) has already been tested [326].

Specific to atherosclerosis, T-lymphocytes-derived MVs bearing sonic hedgehog induce neoangiogenesis and endothelial repair through the NO pathway [327,328]. Of interest, a very recent study has shown that EVs loaded with Smad2/3-siRNA from subcutaneous adipose tissue stem cells and bone marrow MSCs ameliorate vascular dysfunction and regress inflammation-mediated atherosclerosis [329]. Finally, EVs from human placental-expanded stromal cells bear a functional corona with immunomodulatory and angiogenic effects with potential application for peripheral artery disease [330].

Engineering of nanoparticles with encapsulated biological molecules as novel therapeutic vehicles is another emerging option. These nanoparticles can be engineered with specific surface molecules to boost their in vivo stability, specificity, and bioactivity [331,332,333,334]. Among the increasing number of studies in this regard (mainly focused on cancer), it is relevant to highlight the magnetic nanoparticle-based gene transfer into the vascular endothelium in order to tackle the diseased vasculature [335,336,337]. To increase delivery efficiency to specific tissues, EVs have been combined with biomaterial scaffolds (tissue engineering). For instance, loading EC-derived EVs with bioprinted 3D structures [338] or a sustained delivery of EPC-derived EVs from injectable hydrogels [339] to support neovascularization and ameliorate cardiac dysfunction after MI. Recently, a synthetic nanotechnology leveraged to mimic the procoagulant function (adhesion and aggregation) of native platelets on nanoparticles has been reported. Platelet-mimicking procoagulant nanoparticles (PPNs), containing PS on their surface, can resist clot lysis and increase clot stability, leading to reduced bleeding and improved survival in rodent models of thrombocytopenia and traumatic hemorrhage. The design of PPNs able to amplify thrombin and fibrin generation at injured sites and enhance homeostatic clot formation emphasizes the multiple therapeutic applications of engineered EVs [340].

There are already clinical studies registered to use EVs in a wide range of diseases (104 trials listed on ClinicalTrials.gov, US National Library of Medicine, Bethesda, MD, USA). Several parameters need to be further explored before the therapeutic effects of EVs observed in the experimental setting can be translated into the clinics such as administration route (systemic injection, topical or radiologic, ultrasound, or endoscopy-guided administration, intranasal injection, oral treatment), dose, immunogenicity, in vivo biodistribution, half-life, and efficiency of cargo delivery [313]. Additionally, standardized, scalable, reproducible, and efficient protocols for EV production with minimal batch-to-batch product variation are required for future engineered EV-based therapeutics [341].

## 6. Future Perspectives and Conclusions

Extracellular vesicles play essential cell communication roles in cardiovascular biology. Considering the implication of extracellular vesicles in inflammatory atherothrombosis, targeting of the EV-driven innate and immune responses offers new therapeutic avenues. We also pinpointed pathways by which EVs could act as protective factors inducing anti-inflammation and atheroprotection that may serve as potential therapeutic targets against atherosclerosis onset and progression. Further experiments are required in the pathophysiological context, taking into account surrounding cells and spatiotemporal dynamics by using novel imaging techniques [342], omics-based methodology [230], organ-on-chip systems [343,344], or model organisms such as zebrafish [345] to reveal new features of EVs. While challenges remain, EVs emerge as liquid-biopsy-based markers with potential diagnostic and prognostic use in the clinics. Improvements in single-cell and high-resolution flow cytometry will allow better understanding of EVs as biomarkers. Finally, the use of native or bio-engineered EVs might represent novel drug delivery tools to tackle cardiac regeneration, inflammation, and atherosclerosis. We expected that in the near future more preclinical and clinical studies within EVs will help to define the added value of EVs into precision medicine and translate into broader EV-based therapeutic armamentarium for patients with atherosclerotic cardiovascular disease.
